# Description of *Nicrophorus efferens*, new species, from Bougainville Island (Coleoptera, Silphidae, Nicrophorinae)

**DOI:** 10.3897/zookeys.311.5141

**Published:** 2013-06-20

**Authors:** Derek S. Sikes, Tonya Mousseau

**Affiliations:** 1 University of Alaska Museum, 907 Yukon Drive, Fairbanks, AK 99775-6960, USA; 2 Mount Royal University, 4825 Mount Royal Gate Southwest, Calgary, AB T3E 6K6, CANADA

**Keywords:** *Nicrophorus*, Silphidae, burying beetles, taxonomy, Solomon Islands, Papua New Guinea

## Abstract

A new species of *Nicrophorus* in the *nepalensis* species-group, *Nicrophorus efferens* Sikes and Mousseau, is described from Bougainville Island in the Solomon Islands archipelago. It is distinguished from the known species of the genus *Nicrophorus* and its likely closest relative, *Nicrophorus reticulatus* Sikes and Madge, based on external morphology. A comparison among the four *Nicrophorus* species known from the Solomon Island archipelago and Papua New Guinea is presented.

## Introduction

The *nepalensis* species group (Coleoptera, *Nicrophorus*) is the second largest species group within the genus *Nicrophorus*. Species of this group are primarily montane in regions of eastern Asia and the Malay Archipelago, ranging in longitude from 73°E (Pakistan) to 160°E (Guadalcanal) and latitude from 51°N (Ussuri, Russia) to 9°48'S (Papua New Guinea) (Sikes et al. 2006). In the first descriptions of the species group, Portevin (1926), who originally aligned its members, and Hatch (1927), who formally recognized the species group, included five species: *Nicrophorus nepalensis* Hope, *Nicrophorus podagricus* Portevin, *Nicrophorus heurni* Portevin, *Nicrophorus quadripunctatus* Kraatz, and *Nicrophorus maculifrons* Kraatz. Růžička et al. (2000) added two more species to the group, *Nicrophorus montivagus* Lewis and *Nicrophorus sausai* Růžička, Háva, and Schneider (Růžička et al. 2000). Sikes (2003) removed *Nicrophorus sausai*, but added *Nicrophorus apo* Arnett and *Nicrophorus insularis* Grouvelle. The most recent taxonomic and phylogenetic work on the *nepalensis* species group is that by Sikes et al. (2006) who included seven newly described species, bringing the total number of species in the group to 15.

Only two species of *Nicrophorus* were known from the Solomon Islands archipelago prior to this study: *Nicrophorus reticulatus* Sikes and Madge and *Nicrophorus kieticus* Mroczkowski (Sikes et al. 2006). In this work, a third *Nicrophorus* species for the region, from Bougainville Island, Papua New Guinea, is described. This new species is described within the context of an ongoing revision of the subfamily (see Sikes and Peck 2000, Sikes 2003, Sikes 2005, Sikes et al. 2002, 2006, 2008, Mousseau and Sikes 2011).

## Methods

The 6 adult specimens comprising the type series were borrowed from the Bernice Pauahi Bishop Museum of Hawaii (BPBM). One paratype from this series will be deposited in the BMNH. These were compared with specimens from the following collections: FMNH – Field Museum of Natural History, Chicago, IL, USA; DSSC – Derek S. Sikes Collection, University of Alaska Fairbanks, Fairbanks, AK, USA; BMNH – Department of Entomology, The Natural History Museum, Cromwell Rd, London, UK. Four of the type specimens were broken with parts associated on separate pins. All *nepalensis*-group characters were coded independently by both authors and any differences of opinion were discussed to reach a consensus. Morphological data were managed using MacClade 4.04 OSX (Maddison and Maddison 2002) in one NEXUS file. The description was prepared by editing the output generated by use of the ‘export descriptions’ menu option of MacClade. Characters selected are relevant to the subfamily Nicrophorinae so include some characters invariant within the subfamily (synapomorphies of the subfamily) and invariant within the *nepalensis* species group. Also included are absence character states (for example “Frons black, without orange spot” – because an orange spot is common for species in this species group). Specimen data were managed in MANTIS (Naskrecki 2001). Google Earth build 6.1.0.5001 was used to georeference the type locality. The map was prepared using SimpleMappr (Shorthouse 2010). A red determination label bearing a unique alphanumeric code was placed on the pin of each specimen examined. These codes are listed in the material examined data to provide unambiguous association of specimens with data. Habitus photos of *Nicrophorus reticulatus* were captured using a Nikon D100 and a 60mm Nikkor lens at f22, using incandescent and fiber optic lights with a tripod. Beetles were positioned in front of an 18% grey photography card to simplify the exposure. Images of *Nicrophorus efferens* were captured using a Leica DFC425 camera mounted on a Leica MZ16 stereomicroscope in combination with Leica Application Suite © software v.3.8.0; after the specimen had been washed in warm ddH_2_O and Ammonium Hydroxide prior to a ~24h soak in acetone to remove grime and oils thereby brightening the elytral pattern. Images were edited using Adobe Photoshop v.7 to remove the background, stack images of multiple focal planes, and lighten the images. Observations were made with a Leica MZ16 stereomicroscope (7.1x-115× magnification, 1x planapochromatic objective/10x eyepieces, max resolution 420 Lp/mm, Leica Microsystems (Switzerland) Ltd.). Measurements were made using an ocular micrometer in the MZ16 scope. The scanning electron micrograph of the *Nicrophorus reticulatus* elytron was taken with an Environmental SEM (system 2020, version 3.53, FEI company 1999), which, unlike standard SEM, does not require gold-coating of specimens and thus is ideal for imaging type specimens. The SEM image of *Nicrophorus efferens* was taken with a Zeiss (LEO) EVO 60 using variable pressure as an alternative to high vacuum and gold coating.

## Data resources

The data underpinning the analyses reported in this paper are deposited at GBIF, the Global Biodiversity Information Facility, http://ipt.pensoft.net/ipt/resource.do?r=type_specimen_data_for_new_species_nicrophorus_efferens.

## Taxonomy

### Nicrophorus efferens

Sikes & Mousseau sp. n.

urn:lsid:zoobank.org:act:7B4F8F2A-CC50-407A-BD15-CEC84EB706F6

http://species-id.net/wiki/Nicrophorus_efferens

**Figure 1.**
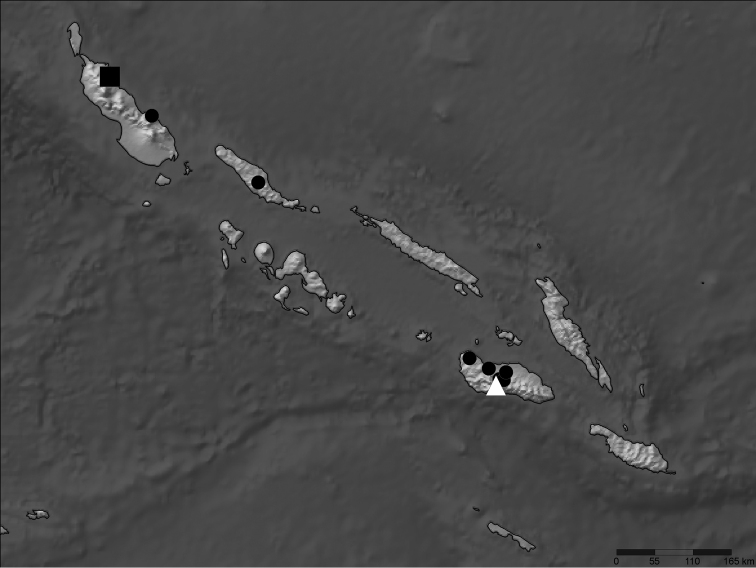
All georeferenced records for *Nicrophorus efferens* (black square), *Nicrophorus kieticus* (black circles) and *Nicrophorus reticulatus* (white triangle) in the Solomon Islands archipelago.

**Figure 2.**
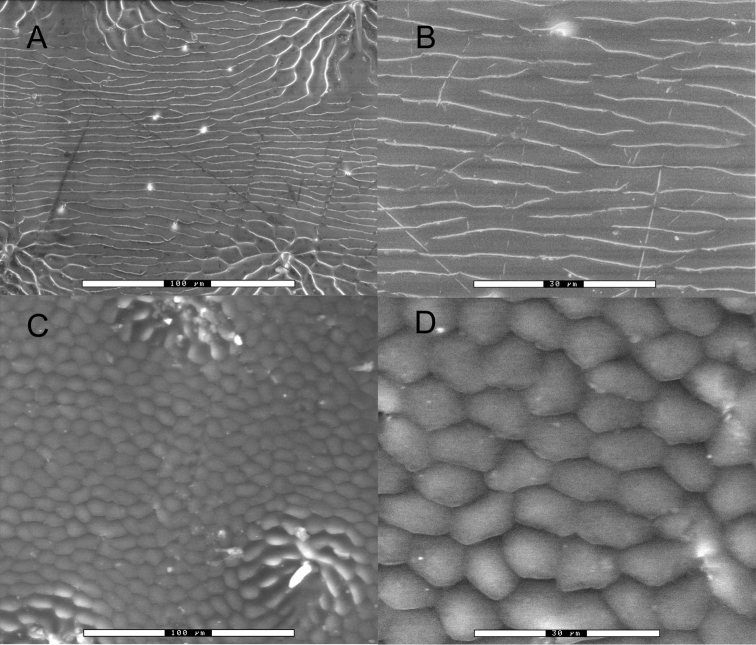
Scanning electron micrographs of elytral microsculpture. **A** *Nicrophorus efferens* paratype BPBM124191Nic, 500×, scale bar is 100 µm **B** *Nicrophorus efferens* 1500×, scale bar is 30 µm **C** *Nicrophorus reticulatus* 500× scale bar is 100 µm **D** *Nicrophorus reticulatus* 1500× scale bar is 30 µm.

**Figure 3.**
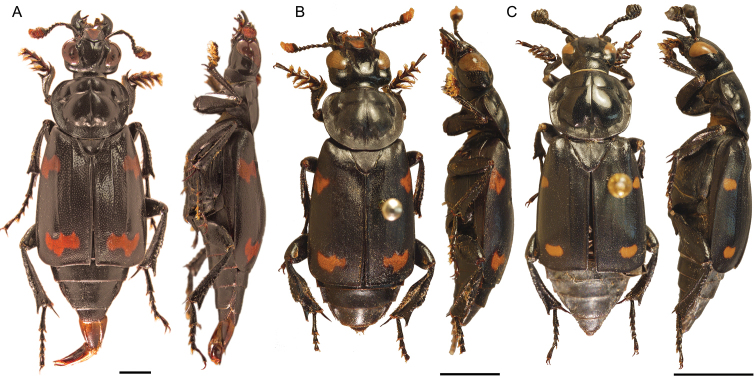
Dorsal and lateral habitus of adult males (at different scales). **A** small male (pronotal width 5.02 mm) with aedeagus everted, *Nicrophorus efferens* (holotype BPBM124189Nic), scale bar is 2 mm, fifth protarsomeres are missing **B** large male (pronotal width 6.6 mm) *Nicrophorus reticulatus* (paratype BMNH000826), scale bar is 5 mm **C** small male (pronotal width 5.19 mm) *Nicrophorus kieticus* (BMNH000809Nic) scale bar is 5 mm.

**Figure 4.**
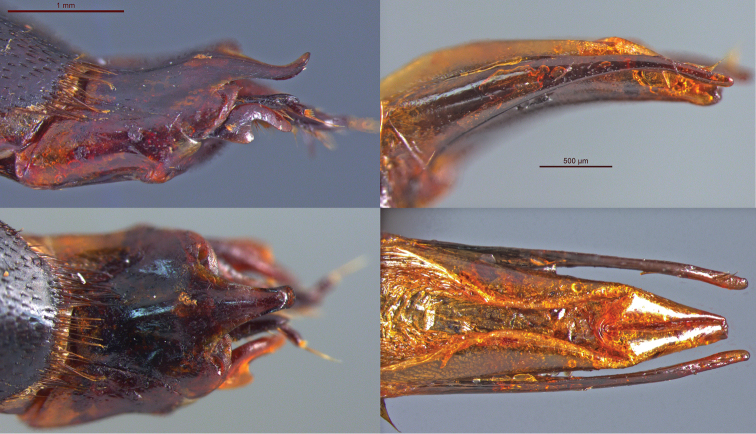
Genitalia. Female ovipositor (paratype BPBM124190Nic), **A** lateral **B** dorsal. Scale bar is 1 mm. Male aedeagus (holotype BPBM124189Nic) **C** lateral **D** dorsal. Scale bar is 500 µm.

Figs 1 , 2A, B , 3 , 4

#### Holotype.

Male (in BPBM), here designated, labeled “Bougainville: NE Mutahi. 700m 18 km S. E. Tinputz” / “8–14. III. 1968” / “Tawi Collector BISHOP” / “Nicrophorus kieticus Mroczkouski [sic] det. S.B. Peck. 1993” / “HOLOTYPE *Nicrophorus efferens* Sikes & Mousseau 2013 BPBM124189Nic” [red paper].

#### Paratypes.

5 Specimens. **2 Females**, labeled “Bougainville: NE Mutahi. 700m 18 km S. E. Tinputz” / “15–21. III. 1968” / “Tawi Collector BISHOP” / “Nicrophorus kieticus Mroczkouski [sic] det. S.B. Peck. 1993” / “PARATYPE *Nicrophorus efferens* Sikes & Mousseau 2013 BPBM124190Nic” [red paper], this specimen’s head is mounted on a card below the body (deposited in London, BMNH), BPBM124191Nic, this specimen’s abdomen (genitalia missing) is mounted on a card on a separate pin and its left elytron is mounted below the body on a card. **1 Female**, labeled “Bougainville: NE Mutahi. 700m 18 km S. E. Tinputz” / “8–14. III. 1968” / “Tawi Collector BISHOP” / “Nicrophorus kieticus Mroczkouski [sic] det. S.B. Peck. 1993” / “PARATYPE *Nicrophorus efferens* Sikes & Mousseau 2013 BPBM124192Nic” [red paper], phoretic mites removed from body into genitalia vial on pin below body. **1 Female**, labeled “Bougainville: NE Mutahi. 700m 18 km S. E. Tinputz” / “1–7. III. 1968” / “& R. Straatman Collectors BISHOP MUSEUM” / “Nicrophorus kieticus Mroczkouski [sic] det. S.B. Peck. 1993” / “PARATYPE *Nicrophorus efferens* Sikes & Mousseau 2013 BPBM124193Nic” [red paper], this specimen’s prothorax and head are mounted on a card below the body. **1 Male**, labeled “Bougainville: NE Mutahi. 700m 18 km S. E. Tinputz” / “1–7. III. 1968” / “Tawi Collector BISHOP” / “Nicrophorus kieticus Mroczkouski [sic] det. S.B. Peck. 1993” / “PARATYPE *Nicrophorus efferens* Sikes & Mousseau 2013 BPBM124194Nic” [red paper], specimen broken and mounted on two pins: hind legs and pygidium on card below body, on pin 2 genitalia in vial with glycerin, head and prothorax on card.

#### Type locality.

[Papua New Guinea]: Solomon Islands [Archipelago]: Bougainville: NE Mutahi, SE Tinputz, 700m, [~ 5.716°S, 155.086°E +/- 2 km, WGS84] (Fig. 1).

#### Measurements.

(2 males, 4 females), pronotal width: male 5.02 – 5.87, 5.45 ± 0.6 mm, female 5.34 – 6.42, 5.82 ± 0.45 mm.

**Diagnosis.** *Nicrophorus* with center of abdominal sternite 3 between metacoxae glabrous, with long erect brown setae lateral of center hidden above and exposed posterior of coxae (shared with only four *Nicrophorus* species: *Nicrophorus distinctus* Grouvelle, *Nicrophorus apo* Arnett, *Nicrophorus heurni* Portevin, and *Nicrophorus reticulatus* Sikes & Madge); epipleura black or black throughout with some dark orange in the posterior dorsal portion (of these four, shared with only *Nicrophorus reticulatus*); elytral disc microsculpturing transverse, straight, narrow with breaks (Fig. 2A, B).

#### Description.

Eight character states not shared with *Nicrophorus reticulatus*, the likely closest relative of *Nicrophorus efferens*, are indicated below by use of italics.

**Head.** Mandibles asymmetrical with dent receptive notch on right mandible. Mandibles preapically wide, short, stout; with dorsal, ridged groove. Maxillary palpus not elongate, concealed beneath mandible when mandibles open. Segment 1 of labial palpus as long as segment 2. Labrum with bilateral pair of elongate setae in clusters. Clypeal membrane orange, yellow or brown, not black. Female clypeal membrane fasciform, male clypeal membrane campanulate and produced posteriorly and laterally enclosed by clypeus. Gula bar of large males reduced to thin strip, replaced by membrane, not contiguous with submentum. Frontoclypeal (epistomal) suture present. Gular sutures confluent, reducing gula to small triangle posterior of gula bar. Antennomeres 2 and 3 fused making an 11 segmented antenna appear 10 segmented. Antennal scape with posterior face flattened (fitting against eyes). Ridge surrounding antennal socket forming distinctly ridged lateral edge of clypeus. Antennal club abrupt, large; basal segment black, apical 3 orange. Posterior of antennal club segments unlike anterior, with large raised ridge, with sharp lateral edges. Basal antennomere of club oval, transverse, not circular. Antennal club segment joints at edge of segments. Antennomeres of club weakly emarginate. Frons black, without orange spot. Epicranial sulci (grooves along inner margin of eyes) long, reaching posterior of eyes and usually joining. Postocular bulge present. Lateral margins of head of large males parallel, or subparallel, giving rear of head a square appearance. Width across postocular bulge of large males less than width across eyes. Post-ocular bulge of large males larger than females. Posterior margin of eye of large males in lateral view sinuate. Width of eyes of large males wide (width greater than or equal to half length). Dorsum of neck with two nonpunctate band(s). Microsculpture of frons absent (smooth, polished).

**Thorax**. Microsculpture of pronotum disc isodiametric. Pronotum of large males orbicular. Anterior margin of pronotum straight or weakly concave. Pronotal anterior impressions complete, with distinct inner arcs. *Setae on posterioventral portion of hypomeron long*, *erect, sparse; filling region extending a third or less length between trochantin and posterior margin, restricted to upper region of sclerite.* Anterior corner of hypomeron glabrous. Triangular depression at midpoint posterior margin of pronotum absent. Pronotum disk black. Posterior of pronotum with flat margin or border. *Setae present on pronotum*, brown to light-brown, short (ca. < 0.1mm), sparse, and restricted to margins. Pair of ‘v’-shaped bumps present on posterior of pronotum. Medial groove of pronotum present. Antemesosternal sclerite cordiform and bilobate. Elytral locking device under apex of scutellum composed of single channel, without distinct, thin, raised median ridge. Humerus immediately dorsal of epipleural ridge black. Humeral setae present, short and not forming row. Epipleuron entirely black or black throughout with some dark orange in the posterior dorsal portion. Posterior epipleural ridge without isolated single-file row of contiguous preapical setae. Epipleural ridge distinct and short, to tip of scutellum. Epipleuron glabrous, or with very sparse, extremely small setae (ca. the size of a puncture). Lateral margin of elytron glabrous, or with very sparse, extremely small setae (ca. the size of a puncture). Elytral surface without long setae. Elytra bicolored and bifasciate. Elytron with costae not distinctly raised but visible to naked eye. Region adjacent to apex of elytral suture black. Profile of elytra in lateral view raised posteriorly. Anterior fascia of elytron without black spot, small, triangular-square, just reaching 2nd costa. Posterior fascia between costae, anterior margin u -shaped between costae 1 and 2 (with bottom of u towards posterior). Posterior fascia not touching lateral or posterior margins of elytron. Elytral posterior fascia without black spot. Elytral posterior fascia greatly reduced, far from suture, jagged. *Elytral microsculpture transverse straight*, *narrow with breaks*. Elytral color varation – greatly increased orange and all black forms unknown. Ventral surface of elytron with golden sheen. Row of setae facing inward or downward on inner lateral margin of elytra in posterior quarter along lateral ridge of elytron in a distinct, single file row. *Posterior margin of elytron with 5-7 clusters of very short, dark setae*. Flange along mesepisternal anterior margin tapering gradually towards mesosternum. *Metanotal subalare with sharply rising*, *internally margined ridge along anteriomedial edge*, anteriormedial ridge narrow leaving a wide depression. Metepimeron constricted. Metepisternum with upper third impunctate and glabrous. Metasternal pubescence medially (between mesocoxae) and laterally, long, *dark brown*. Metasternum with long setae, bald patch posterior of mesocoxae absent. Metepimeral posterior lobe with sparse, short brown setae throughout lobe, or glabrous. Metasternum posterior margin edge glabrous. Furcal arms of metendosternal apophysis directed posteriorly (forming acute angle with main stalk). Laminae of metendosternal apophysis joining furcal arms to anterior projection of stem. Ventral laminae of metendosternal apophysis reaching from anterior projection to bend in main stalk (anterior 3 fourths of entire length). Radial hinge of wing notched. Apex of pterostigma of wing truncate-rectangular.

**Abdomen.** Pair of stridulatory files on tergite 5 present, files parallel, separated by 2 or more file widths, touching posterior margin of tergite. Stridulatory file scraper on venter of elytron near elytral apex (< 0.2mm). First abdominal spiracle slit short, less than one third length of spiracle, in straight line with center line of spiracle. First abdominal spiracle lobed, with small bulb projecting anteriorly. Posterior margins of abdominal segments 3-6 with short setae (2-3 times longer than the distance between adjacent setae). Center of sternite 3 between metacoxae glabrous, but with long erect setae lateral of center under coxae. Spiracle of tergite six elongate, slit-like, and parallel to lateral margin of tergite, or forming less than 30-degree angle with lateral margin. Tergite seven with short depressed setae. *Abdominal setae dark brown*. *Setae of tergite 9 of males (pygidium) brown.*

**Legs.** Venter of protibia apex with lateral process. Anterior face of protrochantin with regions of dense pubescence composed of short, recumbant golden setae. Anterior of procoxae with short setae on basal half. Lateral margin of anterior of procoxae smooth, without ridge or bump. Meso- and metatibia apical angle produced into lobe. Outer margin of mesotibia straight or curved outwards. Inner margin of metatibia of large males gradually curved outwards. Inner margin of metatibia with inner face not forming wide channel entirely filled with dense long setae. Middle of outer margin of metatibia slightly swollen in large males. Middle of inner face of metatibia of large males greatly widened (2.5 or greater than width at base). Metatibia straight. Inner face of apex of metafemora with circular or oval cluster of short setae occupying 1/4 to 1/2 of apical region. Inner face of base of metafemora with single ridge separating medial setose area (area not depressed) from lateral half. Female metafemora slender (length ≥ 2.5 times greatest width). Posterior/ventral face of metafemora flattened with ridged edges. Metatrochanter spine of males short and subapical, apex pointing parallel (or almost parallel) to leg, straight, not recurved dorsally. Metacoxae wider than long. Metacoxae with anterior line complete for half or more of metacoxa. Posterior margin of metacoxae without white microsetae. Tarsal empodium bisetose. Venter of metatarsomere 1 glabrous medially, especially distally, leaving narrow glabrous channel, with dense cluster of long setae at apex.

**Ovipositor.** (Fig. 4A, B) Valvifer with claw. Proctiger (T10) apex strongly lobed, spatulate, not bifurcate. Spatula on proctiger (T10) apex narrow (ca. equal to half the width across widest region of metatarsomere 5). Proctiger (T10) lobe with pubescent apex, ventral setae. Proctiger (T10) venter of spatula apex smooth, lacking medial keel-like ridge. Gonocoxite terminal claw absent. Dorsal ridge on proctiger (T10) absent. Paraproct (T9) apex glabrous, with well-defined, raised ridge. Paraproct without process. Valvifer claw glabrous, dentate; dentition composed of small rounded lobe situated in the middle to distal third of the valvifer.

**Aedeagus.** (Fig. 4C, D). Paramere apex rounded, with setae laterally not reaching the apical curve. Paramere ventral setal patch not composed of 5 evenly spaced setae of identical length and not overlapping with apicolateral patch. Without 3rd paramere setal patch or paramere flange. Parameres not constricted behind apex. Parameres evenly curved, tapering towards apex.

**Variation.** The holotype male and the largest female paratype has an epipleuron mostly black throughout, but with some dark orange faintly visible in the posterior dorsal portion. Two of the type specimens have no setae along the posterior margin of the elytra, presumably they have been lost to abrasion.

**Geographic Distribution.** This species is only known from the type locality (Fig. 1).

#### Etymology.

From the latin verb ‘*effero*’ to carry out for burial, bear to the grave, bury; present participle in the nominative singular.

## Discussion

This new species is likely closely related to one if not both of the *Nicrophorus* previously known from the Solomon Islands archipelago (*Nicrophorus reticulatus* [Figs 2, 3] and *Nicrophorus kieticus*, [Figs 1, 3]) and may be the key to untangling the mystery of how they are related. Two of its three diagnostic characters are likely synapomorphies between it and *Nicrophorus reticulatus*. Although the holotype and one paratype of the four known specimens of *Nicrophorus reticulatus* have entirely black epipleura, the two remaining paratypes have epipleura mostly black throughout, but with a small region of dark orange in the posterior dorsal portion. This weakly present orange region is present on two of the *Nicrophorus efferens* type specimens. These two species, along with *Nicrophorus kieticus*, also share greatly shortened setae along the posterior margins of abdominal sterna 3 to 6 and four highly reduced and similar looking fasciae on the elytra (Fig. 3). However, *Nicrophorus efferens* differs strongly from *Nicrophorus kieticus* in its possesion of most synapomorphies of the *nepalensis* group (Sikes et al. 2006) including orange antennal clubs and an epipleural ridge no longer than the tip of the scutellum. *Nicrophorus kieticus* has black antennal clubs and a longer epipleural ridge, among a total of 12 character states we have found that *Nicrophorus kieticus* does not share with members of the *nepalensis* group. Of 185 morphological characters coded for the subfamily, *Nicrophorus efferens* shares 95.7% of its character states with *Nicrophorus reticulatus*, 92.4% with *Nicrophorus heurni*, a species restricted to the island of New Guinea, and 84.8% with *Nicrophorus kieticus*. The primary character that differentiates *Nicrophorus efferens* from *Nicrophorus reticulatus* is that the former has elytral microsculpturing typical of the *nepalensis* group (Fig. 2A, B) while the latter is the only species of the group with isodiametric microsculpturing (Fig. 2C, D). In the keys of Sikes et al. (2006) *Nicrophorus efferens* would key out as *Nicrophorus reticulatus* or *Nicrophorus heurni* so an additional couplet is necessary to key out this new species. Given the rarity of these species in collections it would not be surprising if more new *Nicrophorus* species await discovery at higher elevations of the Melanesian and Malay Archipelago islands.

**Table:** 

1	Elytra with epipleuron partially or entirely black (Fig. 3A, B)	2
–	Elytra with epipleuron entirely red–orange	4
2	Elytral disc with strongly transverse microsculpturing (Fig. 2A, B)	2.5
–	Elytral disc with isodiametric microsculpturing (Fig. 2C, D)	*Nicrophorus reticulatus*
2.5	Elytra with epipleuron black with entire medial (dorsal) half orange	3
–	Elytra with epipleuron entirely black (or black with some dark orange in the posterior dorsal portion)	*Nicrophorus efferens*

## Supplementary Material

XML Treatment for Nicrophorus efferens
